# Cross-Sectional Associations of Screen Time Activities With Alcohol and Tobacco Consumption Among Brazilian Adolescents

**DOI:** 10.3389/ijph.2023.1605816

**Published:** 2023-07-13

**Authors:** Priscila Cristina dos Santos, Bruno Gonçalves Galdino da Costa, Marcus Vinicius Veber Lopes, Luís Eduardo Argenta Malheiros, Lauren Arundell, Kelly Samara da Silva

**Affiliations:** ^1^ Department of Physical Education, Federal University of Santa Catarina, Florianopolis, Brazil; ^2^ School of Physical and Health Education, Nipissing University, North Bay, ON, Canada; ^3^ Institute for Physical Activity and Nutrition (IPAN), Deakin University, Geelong, VIC, Australia

**Keywords:** screen time, alcohol drinking, smoking, youth, cross-sectional study

## Abstract

**Objectives:** Little is known about the association between specific types of screen time and adolescents’ substance use. Thus, this study aimed to investigate the associations between screen time for studying, working, watching movies, playing games, and using social media and frequency of alcohol and tobacco use.

**Methods:** In this cross-sectional study, Brazilian adolescents answered survey questions related to frequency of tobacco and alcohol consumption, and reported their daily volume of five types of screen time. Multilevel ordered logistic regression models were performed.

**Results:** Each 1-hour increase in ST for studying was associated with 26% lower odds of smoking (OR = 0.74; 95% CI: 0.61–0.90) and 17% lower odds of drinking alcohol (OR = 0.83; 95% CI: 0.76–0.91) in the past 30 days. The increase of 1 hour of social media use was associated with 10% greater odds of smoking (OR = 1.10; 95% CI: 1.02–1.18) and a 13% greater chance of consuming alcohol (OR = 1.13; 95% CI: 1.08–1.18) in the past 30 days.

**Conclusion:** The association between screen time and substance use appears to be type-specific. Future longitudinal research is needed to explore causal relationships.

## Introduction

Scientific evidence regarding the adverse effects of alcohol and tobacco consumption among children and adolescents has been accumulated over the course of several decades [1–3]. This evidence consistently establishes a correlation between these behaviors and social issues [1], chemical and psychological addiction [1, 2], as well as hindered development of the nervous system [2]. In light of this scientific consensus, numerous countries have implemented prohibitions on the use of alcohol and tobacco by adolescents, have enacted laws and policies, and have promoted interventions aimed at preventing their experimentation and consumption [3]. Furthermore, legislation has been enacted to limit the marketing of these substances to young individuals. Additional measures, such as the imposition of higher taxes and the prohibition of commercials and other marketing strategies on mainstream television programs targeting youth, have also been implemented [3]. Consequently, there has been a significant decline in alcohol and tobacco use among adolescents in many countries in recent decades [4]. Nevertheless, studies indicate that adolescents continue to engage in the use of these substances in various regions, with a global prevalence rate of 17.0% for tobacco use [4] and 26.5% for alcohol consumption among individuals aged 15 to 19 years [5]. In Brazil, the most recent national school health survey revealed prevalence rates of 6.8% for tobacco use and 28.1% for alcohol consumption within the past 30 days [6].

Screen time (ST) encompasses various activities performed on screen-based devices, including watching television, engaging in social media on smartphones, playing video games on consoles, or utilizing tablets for educational purposes [7]. Digital media use and screen time have been identified as risk factors for tobacco and alcohol use among young individuals [8]. Numerous studies have established a connection between the duration of television viewing, computer use, and video game playing and an increased likelihood of tobacco and alcohol consumption among youth [9–11], as well as associations between alcohol and tobacco references via screens (e.g., through social media and video games) and alcohol and tobacco use/experimentation [12–15]. Considering the ever-evolving nature of technology and its influence on youth, the relationship between novel and contemporary forms of screen time and alcohol and tobacco use among young individuals is still being explored, and further evidence in this area is continuously emerging.

In Brazil, there is a significant prevalence of high screen time (ST) among young individuals, as evidenced by a study comparing children and adolescents from 12 countries, which found Brazil to have the highest rate of non-compliance with current guidelines of limiting recreational ST to less than two hours per day [16]. Recent data from the Brazilian population indicates that 36% of adolescents spend two or more hours watching television daily, and when considering sitting time, which encompasses ST as well, 53.1% of adolescents spend more than three hours engaged in this behavior [6]. This is particularly worrisome considering that excessive ST has been linked to adiposity, unhealthy dietary patterns, depressive symptoms, and diminished quality of life among children and adolescents, as highlighted in a recent review of systematic reviews [17]. Previous studies examining the relationship between ST and health outcomes, as well as the use of tobacco and alcohol, have certain limitations. They primarily focused on quantifying time spent using specific devices, such as televisions or video games, without specifically identifying the activities undertaken during that time (e.g., play games, watch videos, and use social media). With advancements in technology, especially with the proliferation of mobile smart devices, screen activities have become less device-dependent. For instance, smartphones allow users to watch videos, play games, and access social media platforms [18]. Additionally, previous studies have often overlooked ST indicators for non-recreational purposes, such as studying and working [17, 18] which may have both favorable and unfavorable health outcomes [19]. However, its association with the use of alcohol and tobacco remains largely unexplored.

Considering the evolving patterns of technology use and ST activities among youth, the relationship between their engagement in such behaviors and alcohol and tobacco use may diverge from previous study findings, necessitating novel investigations to inform tailored policies and interventions [18]. Furthermore, it is important to acknowledge that the majority of scientific evidence pertaining to ST and its association with health outcomes, as well as alcohol and tobacco use, is derived from studies conducted in high-income countries and may not be directly applicable to the context of low- and middle-income countries. To advance the global agenda regarding ST recommendations, it is crucial to conduct more population-based studies in low- and middle-income countries [20]. Therefore, this study aimed to investigate the associations of ST for studying, working, watching movies, playing games, and using social media with substance use in adolescents.

## Methods

### Study Design and Population

This cross-sectional study used the most up-to-date data (baseline) from the Estudo Longitudinal de Estilo de Vida em Adolescentes (Longitudinal Study of the Lifestyle of Adolescents - ELEVA). The ELEVA Project is a longitudinal study focused on identifying possible changes in health-related lifestyle indicators throughout high school integrated with professional education amongst a sample of Brazilian adolescents. The study population is composed of students of the three Institutos Federais de Educação Tecnológica de Santa Catarina (IFSC) enrolled in the high school with integrated professional courses in the Grande Florianopolis mesoregion, Brazil. In the integrated course, the student who completed elementary school takes technical training at the same time as attending high school. The integrated courses last four years, one year longer compared to the traditional high school curriculum. Frequently, students begin at the ages of 14. The curriculum includes courses of high school level, such as mathematics, languages, and sciences, and specific courses for the professional degrees, that may for example, include advanced chemistry or technical design.

Students from all 52 classes in the three participating schools were invited to participate in the study. The inclusion criteria were the adolescents being enrolled in any of the first-to-third year of high school. The exclusion criteria were having a disability or injury that prevented participation in the collection of study variables. As students from the fourth year were expected to graduate at the end of the year, they would not be included in the repeated measurements and, thus, were not eligible.

Data collection was conducted between August and December 2019. A total of 1,269 adolescents were at school during data collection and were invited to participate. Students who wanted to participate were given informed assent forms, a consent form for their parents or legal guardian to sign, and were required to give assent prior to data collection. In total 1,010 students returned the forms signed and were able to take part in the study. The project was approved by the National Research Ethics System (protocol number: 3.168.745).

### Measurements

All variables used in the present study were collected through an online questionnaire (delivered via SurveyMonkey®), which were completed on devices during school time (e.g., tablets, smartphones, laptops) provided by the research team or students’ personal device. The following questions were used to assess, respectively, tobacco and alcohol use: “During the past 30 days, on how many days have you smoked cigarettes?; During the past 30 days, on how many days did you have at least one drink containing alcohol?” These questions are from the National School Health Survey (PeNSE) [21]. Due to the low number of observations among categories of tobacco use, frequencies were grouped into three categories (0 days; 1–5 days; 6 or more). The same grouping was applied to alcohol use for consistency.

The ST activities were measured using the Questionnaire for Screen Time (QueST) [22], which is a questionnaire composed of the following items: 1) activities related to studying and doing homework; 2) work/internship-related activities; 3) watching videos such as series, movies, news, and sports; 4) playing electronic games; 5) using social media or chat applications. In the questionnaire there is one question for each construct that asks “On a normal weekday/weekend day, how much time do you spend [studying, watching video lessons, reading, doing research or school work] on your computer, television, tablet, cell phone or other electronic device?” The answer options were continuous (hours and minutes) and were asked for both week and weekend days. For the present study, average daily use was calculated by using the following formula: [volume on weekdays*5 + volume on weekend days*2]/7. Given that the ST variables have increased measurement error among participants who reported high volumes of ST, variables were truncated to the 95% percentile. Finally, the volume in each ST was categorized as less than 2 h (˂2 h), 2 h up to 3 h and 59 min (2 h to ˂4 h) and 4 h or more (≥4 h) in line with previous studies analyzing the QueST variables [23]. The QueST demonstrated satisfactory content validity attested by the experts and adolescents through analysis of Content Validity Indexes (CVI) for clarity and representativeness [23]. The reliability of the categorized variables was analyzed within the same sample of the original validation study [22] yielding Gwet’s AC_2_ agreement coefficients ranging from 0.54 to 0.82 for weekdays, and 0.56 to 0.87 for weekend days [23].

Sex (male and female), age (completed years) and the socioeconomic status were evaluated. Socioeconomic status (SES) was determined using a continuous score defined by the Brazilian Association of Research Companies (ABEP). The score is estimated based on ownership of a list of household items (e.g., bathrooms, computers, cars), the highest education level among family members, and living conditions. This score range between 0 and 100, with higher values indicating higher SES.

### Statistical Analysis

For the description of the sample, means and standard deviations were calculated for continuous variables; and absolute and relative frequencies were determined for categorical variables. ST variables were described as both numerical (hours per day) and categorical (two-hour bins). Inferential analyses were performed with ST as continuous variables (hours/day). Given the ordered levels of the outcomes (i.e., tobacco and alcohol), proportional odds logistic regression models were applied to examine their associations with each screen-time use variable (i.e., studying, working, watching videos, playing video games, using social media). Separate models were performed for each outcome and also for each exposure. The multilevel structure of the data was accounted for by estimating random intercepts for classes (level 2) and schools (level 3). The variables sex, age and SES were included as covariates among the fixed effects. The associations were examined separately for each exposure and outcome (ten models in total). The proportional odds assumption was tested using the Brant Test. An exploratory sensitivity analysis was undertaken by including two-way interaction terms between exposures and sex, The interaction terms were not significant at *p* < 0.05 and therefore results are presented for the whole sample. Results were expressed as odds ratio (OR), respective 95% confidence intervals (CI) and less than two hours was the reference variable (exposure). All statistical procedures were conducted in Stata, version 14.2 (StataCorp LP., College Station, TX, USA).

## Results

**FIGURE 1:**
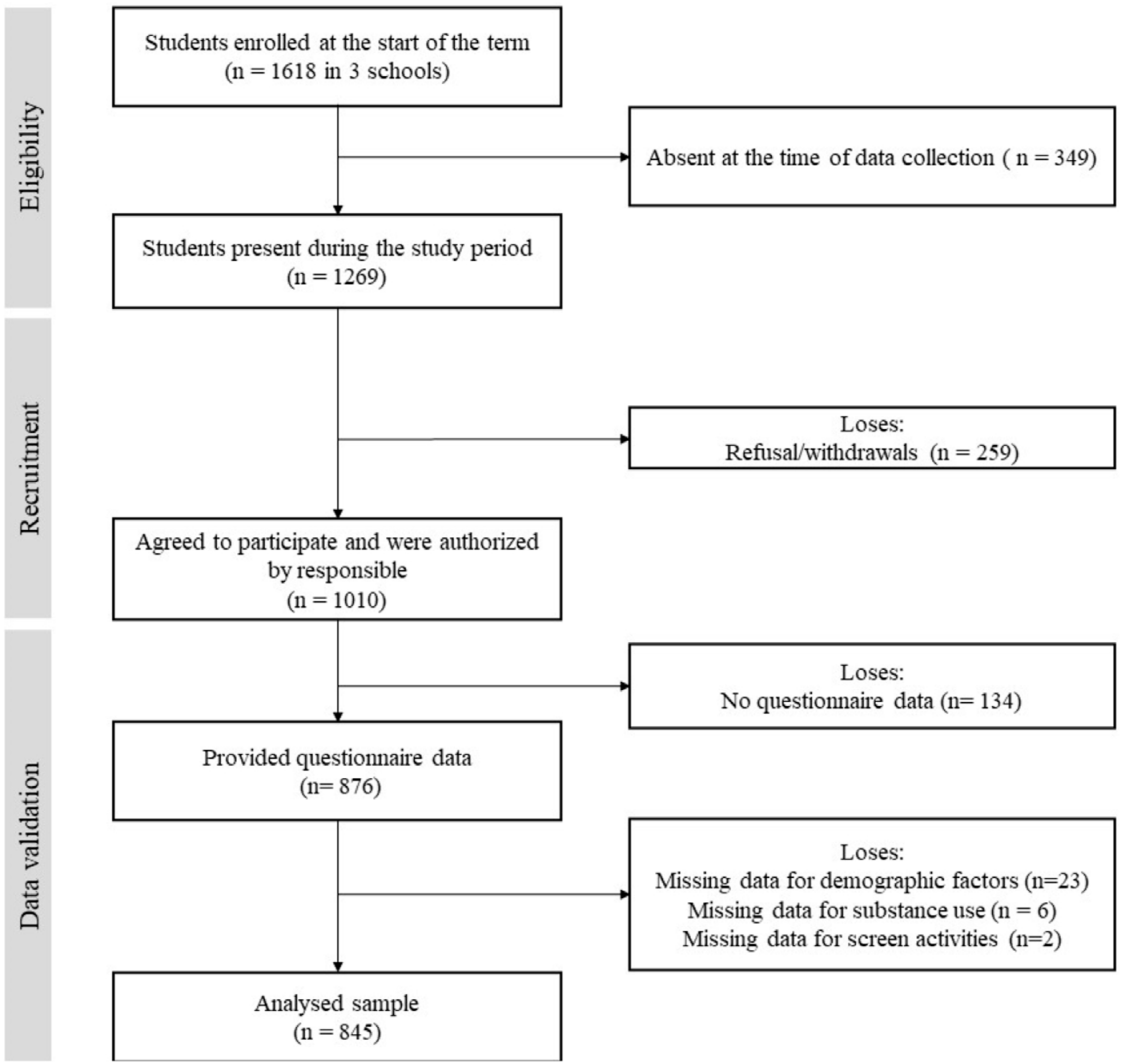
Flowchart diagram of study participants. Longitudinal Study of the Lifestyle of Adolescents, Brazil, 2019.

Of the 1,010 participants who provided consent, 876 completed the survey (86.7%). Of the 876 participants who answered the questionnaire, 845 provided complete data for the relevant variables and were included in the analyses (96.5%). Details can be seen in the flowchart (Figure 1). The sample was comprised of 50.3% females, the mean age was 16.4 (±1.1) years old and the mean SES (0–100) was 39.3 (± 9.9).

The most prevalent screen activity in the four hours or more category was social media (36.8%), while the least frequent screen activity was use for work (4.6%). Alcohol consumption was the most frequent substance used in the past 30 days (43.4%), while 8.2% of the sample used cigarettes in the past 30 days (Table 1). Few adolescents did not use a screen to study (5.8%), 24% did not use a screen to work, 2.3% did not watch videos, 32.5% did not play games and 2.4% did not use social media.

**TABLE 1:** Participant’s characteristics (*n* = 845). Longitudinal Study of the Lifestyle of Adolescents, Brazil, 2019.

Variables	Statistics
Sex, n (%)	
Male	420 (49.7)
Female	425 (50.3)
Age (years), mean ± SD	16.4 ± 1.1
Socioeconomic status (0–100), mean ± SD	39.3 ± 9.9
Screen use for studying (hours), *n* mean ± SD	2.2 ± 2.3
Screen use for studying, *n* (%)	
˂2 h	508 (60.1)
2 to ˂4 h	211 (25.0)
≥4 h	126 (14.9)
Screen use for working (hours), *n* mean ± SD	0.8 ± 2.2
Screen use for working, *n* (%)	
˂2 h	707 (83.7)
2 to ˂4 h	99 (11.7)
≥4 h	39 (4.6)
Screen use for videos (hours), *n* mean ± SD	3.1 ± 2.8
Screen use for videos, *n* (%)	
˂2 h	313 (37.0)
2 to ˂4 h	322 (38.1)
≥4 h	210 (24.9)
Screen use for gaming (hours), *n* mean ± SD	2.0 ± 3.2
Screen use for gaming, *n* (%)	
˂2 h	576 (68.2)
2 to ˂4 h	142 (16.8)
≥4 h	127 (15.0)
Screen use for social media (hours), *n* mean ± SD	4.2 ± 4.3
Screen use for social media, *n* (%)	
˂2 h	286 (33.8)
2 to ˂4 h	248 (29.3)
≥4 h	311 (36.8)
Tobacco use in the past 30 days, *n* (%)	
0 days	775 (91.7)
1–5 days	42 (5.0)
6 or more	28 (3.3)
Alcohol use in the past 30 days, *n* (%)	
0 days	477 (56.4)
1–5 days	269 (31.8)
6 or more	99 (11.7)

SD, Standard Deviation.

The associations between ST activities and substance use are presented in Table 2. Each one-hour increase in ST for studying was associated with 26% lower odds of moving from zero days to 1–5 days of smoking, and from 5 or less days to 6 or more days. Each additional hour of ST for studying was also associated with 17% lower odds of consuming alcohol for more days over the last 30 days. Regarding the use of social media, each additional hour of use was associated with 10% greater odds of moving from zero days to 1–5 days of smoking, and from 5 or less days to 6. In addition, each additional hour of social media use was associated with a 13% greater chance of consuming alcohol for more days over the last 30 days.

**TABLE 2:** Associations between screen time use and tobacco and alcohol consumption (*n* = 845). Longitudinal Study of the Lifestyle of Adolescents, Brazil, 2019.

Exposure	Tobacco use	Alcohol use
	OR (95% CI)	OR (95% CI)
Screen use for studying	**0.74 (0.61, 0.90)**	**0.83 (0.76, 0.91)**
Screen use for working	0.98 (0.78, 1.22)	1.06 (0.94, 1.18)
Screen use for videos	0.02 (0.90, 1.16)	1.01 (0.95, 1.09)
Screen use for gaming	1.05 (0.92, 1.20)	1.04 (0.97, 1.12)
Screen use for social media	**1.10 (1.02, 1.18)**	**1.13 (1.08, 1.18)**

Bold values are significant at *p* <0.05. Models were adjusted by sex, age and socioeconomic level; OR, Odds Ratio; CI, confidence interval.

## Discussion

The purpose of this study was to examine the associations between ST activities and substance use among Brazilian adolescents. Our results showed that ST for studying was associated with lower odds of smoking and consuming alcohol, while using social media was associated with higher odds of smoking and consuming alcohol. However, ST for watching videos and playing games were not associated with substance use. Taken together these results provide further evidence that the relationship between ST and the consumption of substances varies by activities, and efforts to prevent substance use could benefit on focusing on social media use.

According to our results, the use of social media is associated with higher odds of consuming tobacco and alcohol more often. Social media is a digital tool that is widely used by adolescents and enables social interactions through creating and sharing different contents, such as texts, pictures, sounds, and videos. Recent research has broadened our understanding of the possible consequences of indiscriminate use of social media has on adolescents’ health [24, 25]. Although only few comparable studies are available in the literature [8], similar results were observed [10, 24, 25]. Our results corroborate with a study of 10,072 Canadians (11–20 years old) which found that adolescents who visited social networking sites daily were more likely to have drunk alcohol than those who accessed such sites infrequently or those who did not visit them [24]. These results may be related to the content adolescents are exposed to on social media. Due to the omnipresence of these social platforms in the lives of many adolescents, this platform has become a promising place for product advertising which may be responsible for the association of time on social media with alcohol [26] and tobacco use. A systematic review suggested that there is a positive relationship between alcohol marketing exposure and alcohol use behaviors [3]. A study conducted with a national sample of adolescents (aged 14–17) from the United States found the more frequently adolescents saw e-cigarette messages on Instagram and Snapchat, the more positive their attitude toward e-cigarette use [27].

Thus, despite efforts to reduce the presence of alcohol and tobacco-related content on television, alternative platforms such as online streaming services like YouTube continue to feature such content, which is accessible to young individuals [12, 13]. Research has found associations between alcohol and tobacco references in video games and use/experimentation, an association which was observed as well with content on social media platforms like Twitter and Facebook [14]. For instance, a study conducted with college students demonstrated a link between alcohol references on Facebook and alcohol-related issues [14], while another study revealed that several popular video game titles among UK youth contained alcohol and tobacco content and playing these games also correlated with higher rates of alcohol and tobacco experimentation among youth [15]. Considering the ever-evolving nature of technology and its influence on youth, the relationship between novel and contemporary forms of screen time and alcohol and tobacco use among young individuals is still being explored, and further evidence in this area is continuously emerging. It may therefore be important that policies that identify and restrict advertising posts and videos with alcohol and tobacco on social media be developed and implemented, particularly amongst adolescent audiences.

Social media platforms may also be a path to amplify peer pressure, which can also explain the relationship of this particular ST activity with substance use. Exposure to social media content showing a positive view of substance use contributes to the development of favorable attitudes towards substance use [16]. For example, a four-year longitudinal study of 3,612 Canadian adolescents (12.7, SD = 0.5 years) observed that social norms towards alcohol use (i.e., think it is OK for an adolescent to try drinking alcohol to see what it’s like) mediated the association between social media use and alcohol use. The likelihood of adolescents commencing drinking alcohol is greater if they associate drinking with their peers and if they have tolerant attitudes toward deviance or approval of alcohol use [28]. In contrast, social media widely used by adolescents has previously been shown as an effective tool to disseminate important information from a public health perspective. For example, an e-learning format/social media channels program showed an increase in adolescents’ self-efficacy to develop counterarguing and critical thinking skills in response to advertisements [29]. These contrasting associations may be explained by what is called reinforcing spirals, which is the term that describes when people seek out and select content, and to some degrees are exposed to similar content that is consistent with their beliefs, cognition, and preferences [30]. Previous studies among adolescents have shown that reinforcing spirals were observed in the context of violent content and aggression (e.g., people watch violent movies) [31], and political information (e.g., social media reinforces an already established political stance) [32]. Therefore, the development of public policies enforcing an anti-substance use messages on social networks may help adolescents understand the risk they pose to their health and reduce normative perceptions.

The results of the present study on ST related to studying and doing schoolwork showed that each hour spent on these activities was associated with lower odds of consuming tobacco and alcohol more often in the past 30 days. This highlights that there are some positive health outcomes associated with ST when in the context of studying [33]. Studying, even when using screens, has been associated with better academic performance and positive school behavior [34]. And this association can be bidirectional. A longitudinal study with adolescents in Finland, for example, showed that poor academic achievement predicted cigarette experimentation [35]. The results show the importance of investigating beyond overall/combined ST and instead exploring the different ST types (i.e., for game, social media, study, etc.) that can be performed by adolescents, since these ST types have different impacts on adolescents’ behavior and health [36]. In addition, professionals, teachers, and policymakers can encourage teenagers to use screens for study, replacing other activities such as the use of social media.

Our results show that watching videos and playing games with a screen were not related to substance use, which contrasts with previous studies that have shown an association between adolescents’ excessive television time (more than 2 h per day) and the use of substance [37, 38]. Presently, adolescents spend more of their time on streaming platforms and YouTube than watching television [8, 12]. Compared to television, these platforms may have varying levels of alcohol advertising and alcohol-related social norms, which are considered a mediator of alcohol consumption [39]. In contrast, streaming services and video platforms such as YouTube have policies that restrict access content that is inappropriate for underaged individuals, which does not happen on television channels.

In addition, the current study found no association between playing video games and substance use. Studies on the relationship of video games with substance use still have convergences in the literature, and the duration that the Adolescents play [40] and/or gaming addiction [41] may change the association. Problematic use of videogames, which includes addiction-related characteristics, is positively associated with substance use [41]. The relationship with gaming time is mixed, as low use (1 to 5 hours/week) may be protective against substance use (compared to non-users) [40], while elevated use is associated with tobacco, alcohol, and cannabis dependence [40].

The strengths of the present study include the survey instrument which was capable of identifying the activity performed on the screens, where most studies only consider the time in screen devices. The conduct of this study in a middle-income country is extremely relevant as approximately 90% of adolescents in the world live in low- and middle-income countries. The generalizability of findings cannot be extrapolated to the entire population of school adolescents in Brazil, as our sample were from one particular region of the country, with its own particular environmental characteristics, social norms, and culture. Also, the specificity of the schools participating in the study differ from most schools in the country, however this can also be considered a strength as this is a population that is often not evaluated in research.

One of the limitations of the current study is the cross-sectional design that limits causal inference. Although the ELEVA project is longitudinal, only information from the 2019 data collection is available to date. Baseline data collection was conducted in 2019 and another two data collection waves were planned for 2020 and 2021. However, due to the COVID-19 pandemic, data collection in the next years was cancelled. The project has been updated after the pandemic (2022–2023), but data is not yet available. Additionally, the use of self-reported measures is prone to memory and social desirability biases. The reliability of the QueST is not considered optimal, and there is uncertainty about the accuracy of the reported ST. However, the low reliability for some ST variables was expected as not all ST engagement is planned [22]. The research database encompasses a limited set of measured variables that could serve as covariates, potential confounders or effect modifiers in the regression models. Moreover, there may exist bi-directional relationships between screen activities and substance use, which necessitate further examination through longitudinal studies. Given the rapid increase in screen use over the last decade among teenagers, more research is needed to understand the impact of screen content (what social media is accessed, what type of game is played, etc.) on health and well-being.

 In conclusion, the use of screen for studying purposes was associated with lower odds of alcohol and tobacco consumption in the past 30 days . However, higher time spent of social media was associated with higher consumption of both substances. The results support the need for interventions and policies to focus on how much time adolescents spend on screens as well as the type of activity they are performing. However, there is a need for further longitudinal studies to confirm these relationships. Restricting access to substance-use content in social media and/or promoting a reduction in overall social media use may be effective strategies to decrease substance use among adolescents.
